# Vaccine Effectiveness Estimates, 2004–2005 Mumps Outbreak, England

**DOI:** 10.3201/eid1301.060649

**Published:** 2007-01

**Authors:** Cheryl Cohen, Joanne M. White, Emma J. Savage, Judith R. Glynn, Yoon Choi, Nick Andrews, David Brown, Mary E. Ramsay

**Affiliations:** * London School of Hygiene and Tropical Medicine, London, United Kingdom; † Health Protection Agency Centre for Infections, London, United Kingdom; 1 Current affiliations: National Institute for Communicable Diseases, Johannesburg, South Africa, and University of the Witwatersrand School of Public Health, Parktown, South Africa

**Keywords:** Measles, mumps, rubella vaccine, mumps, vaccine effectiveness, outbreak, England, research

## Abstract

The United Kingdom and United States have recently experienced large outbreaks of mumps, which raises concerns about vaccine effectiveness. The effectiveness of the mumps component of the measles, mumps, rubella (MMR) vaccine was estimated using the screening method. In England from January 2004 through March 2005, 312 cases of mumps were reported in children eligible to have received 2 doses of MMR vaccine. Of these children, 52 (16.7%) had received 1 dose of MMR vaccine, and 97 (31.1%) had received 2 doses. Vaccine effectiveness was 88% (95% confidence interval [CI] 83%–91%) for 1 dose and 95% (95% CI 93%–96%) for 2 doses. The effectiveness of 1 dose declined from 96% (95% CI 81%–99%) in 2-year-olds to 66% (95% CI 30%–83%) in 11- to 12-year-olds, and the effectiveness of 2 doses declined from 99% (95% CI 97%–99.5%) in 5- to 6-year-olds to 86% (95% CI 74%–93%) in 11- to 12-year-olds (p<0.001 for 1 or 2 doses). Waning immunity may contribute to mumps outbreaks in older vaccinated populations.

## Abstract 2

As vaccinated children approach adolescence, immunity wanes, which may contribute to outbreaks.

In October 1988, immunization against mumps was introduced in the United Kingdom as a single dose of measles, mumps, rubella (MMR) vaccine offered to all children 12–15 months of age (*1*). Reports of disease declined 79% in the first year, from 20,713 in 1989 to 4,277 in 1990. In 1996, a second dose of MMR was introduced at school entry (*2*). From 1990 through 2003, the number of reported cases remained <5,000 per year (*3*), and since 1995, a high proportion of clinically diagnosed cases were shown by laboratory investigation not to be genuine mumps (*4*).

During 2004–2005, a major increase in reported and confirmed cases occurred in all regions of England and Wales. In 2005, >56,000 clinical cases were reported, most in patients 19–23 years of age (*5*). Of the confirmed cases, <3% occurred in children eligible to have received 2 doses of MMR vaccine routinely (i.e., those born from 1993 through 1999) (*5*,*6*).

During 2005–2006, a large outbreak of mumps involving >2,500 possible cases from 11 states was reported in the United States (*7*). The reemergence of mumps in countries that had high levels of vaccine coverage for many years raises questions about the effectiveness of the mumps component of the MMR vaccine and the possible contribution of waning immunity.

In early clinical trials, the efficacy of a single dose of mumps vaccine was >95%, but estimates of the effectiveness in field evaluations have been 62%–85% (*8*–*18*). The possibility of waning immunity has been suggested in several studies but not conclusively demonstrated (*11*–*13*,*19*). The UK outbreak offered the opportunity to evaluate the effectiveness of 1 or 2 doses of MMR vaccine and to investigate the presence of waning immunity by using routinely collected data.

## Methods

We reviewed clinically reported mumps cases in England from January 1, 2004, through March 31, 2005, that were confirmed by oral fluid mumps-specific immunoglobulin M testing within 6 weeks of symptom onset (*20*). Only case-patients eligible to have received 2 doses of MMR vaccine through the routine program (i.e., those born after 1992) were included. Case-patients 3–5 years of age were excluded because children receive the second dose of MMR between these ages and, therefore, reliable population coverage data are not available for comparison. Possible vaccine-associated cases (i.e., symptom onset within 6 weeks after vaccination) were also excluded. Vaccination histories were obtained from physician records or child health computerized recordkeeping systems.

Quarterly population vaccine coverage data for children 2 and 5 years of age were obtained from the Cover of Vaccination Evaluated Rapidly program (*21*,*22*). Because of changes in health service boundaries and child health computerized recordkeeping systems, data for a small number of areas were missing for some quarterly periods. In addition, 5-year coverage data have been shown to underestimate the true coverage in some areas (*23*). To compensate for this, coverage data were adjusted. Missing values were estimated using linear interpolation from the values submitted in previous and subsequent quarters. When coverage of first-dose MMR for the same birth cohort was lower at 5 than at 2 years of age, the coverage at 5 years of age was assumed to be 3% higher than the value at 2 years of age (based on data from a sentinel surveillance scheme) (*22*). The adjusted data were used to approximate population coverage for school-year cohorts (born from October 1 through September 30 of the following year) to allow for similar levels of exposure within school years. Coverage data at 5 years of age for children born in 2000 were not yet available, so values were estimated from the previous cohort and data from the first 2 quarters. Coverage data were divided into 2 categories, within and outside London, because coverage estimates are lower in London but fairly similar in the rest of the country (*24*).

### Statistical Analysis

Vaccine effectiveness was calculated from the proportion of confirmed mumps cases vaccinated (PCV) and the proportion of the population vaccinated (PPV) using the following formula (*25*): 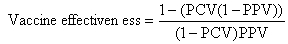


The data were grouped by school year, age, sex, and area of residence (within or outside London) and were analyzed by using logistic regression with an offset (incorporating expected PPV by area and birth cohort) for vaccine coverage. When estimating vaccine effectiveness for 1 dose, those who had received 2 doses were excluded from the calculation of PPV. Similarly, those with only 1 dose were excluded when estimating vaccine effectiveness for 2 doses. To estimate overall effectiveness, a model including only a constant was fitted. Effectiveness in different subgroups was estimated from univariable models. If >1 variable was statistically significant on univariable analysis, multivariable analysis was performed.

Data from computerized child health systems, used for scheduling and recording vaccinations given, have consistently been shown to underestimate vaccine coverage (*26*,*27*). Sensitivity analyses explored the effects of possible underestimation of vaccine coverage by repeating calculations assuming that PPV was 1%, 2%, and 5% higher than that reported. Vaccine effectiveness estimates were also calculated using the unadjusted coverage data to examine the effect of data cleaning.

Estimating coverage in older children from a measurement at 5 years of age could lead to an underestimate of effectiveness if there were a subsequent increase in coverage. Therefore, we obtained data on children vaccinated with MMR after 5 years of age from the child health computer system in the former South Thames region in 2000. Analyses explored the effect of an increase in coverage of 0.04%–0.4% per year of age after 5 years of age. The proportion of persons predicted to be susceptible to mumps, by age in England in 2005, was calculated by multiplying age-specific estimates of vaccine effectiveness by annual birth cohort coverage data.

## Results

### Vaccine Effectiveness

We found 312 confirmed cases of mumps that were eligible for inclusion. Vaccination history was obtained for all case-patients. The proportion of unvaccinated case-patients decreased with increasing age and was higher in younger birth cohorts (Table 1). The proportion of vaccinated case-patients did not differ according to area of residence or sex.

**Table 1:** Characteristics of reviewed mumps case-patients by number of doses of measles, mumps, rubella vaccine received

Characteristic	No. doses received (%)				p value*
	0	1		2	
Age group, y					
2	11 (84.6)	2 (15.4)	–		–
5–6	51 (83.6)	5 (8.2)	5 (8.2)		<0.001†
7–8	52 (61.2)	11 (12.9)	22 (25.9)		–
9–10	35 (39.8)	16 (18.2)	37 (42.1)		–
11–12	14 (21.5)	18 (27.7)	33 (50.8)		–
Birth cohort‡					
1993–1995	31 (30.1)	25 (24.3)	47 (45.6)		<0.001†
1995–1997	42 (48.8)	12 (14.0)	32 (37.2)		–
1997–1999	45 (66.2)	8 (11.8)	15 (22.1)		–
1999–2001	34 (81.0)	5 (11.9)	3 (7.1)		–
2001–2003	11 (84.6)	2 (15.4)	–		–
Area of residence					
Outside London	143 (51.3)	46 (16.5)	90 (32.3)		0.1
London	18 (64.3)	6 (21.4)	4 (14.3)		–
Data not available	2 (40.0)	–	3 (60.0)		–
Sex					
Female	63 (48.8)	19 (14.7)	47 (36.4)		0.2
Male	100 (55.0)	32 (17.6)	50 (27.5)		–
Data not available	–	1 (100.0)	–		–
Total	163 (52.2)	52 (16.7)	97 (31.1)		–

*Fisher exact test. †Children <2 years of age and 2001–2003 birth cohorts excluded for age group and birth cohort, respectively. ‡Birth cohorts for 2 consecutive school years (i.e., children born October 1–September 30 of the following year).

Age at first dose of MMR vaccine ranged from 10 months to 5 years 9 months and at second dose from 16 months to 6 years. Of those who had received MMR, >90% received the first dose at 12–24 months of age and the second dose at 3–5 years of age.

Adjusted population vaccine coverage declined during the study period (Table 2). Outside London, the percentage of children who received 1 dose of MMR by their second birthday declined from 92.7% to 82.7% from the 1993 to the 2002 birth cohort. The percentage who received 2 doses by their fifth birthday was more stable, declining from 78.1% to 76.4% from 1993 to 2000. In London, estimates of coverage were lower and also declined over time.

**Table 2:** Estimated coverage of measles, mumps, rubella vaccine at 2 and 5 years of age by area and birth cohort

Birth cohort*	% Coverage at 2 years of age		% Coverage at 5 years of age			
	Outside London	London	Outside London		London	
	1 dose	1 dose	>1 dose	2 doses	>1 dose	2 doses
1993–1994	92.7	77.1	95.7	78.1	88.6	57.1
1994–1995	92.0	76.6	95.0	78.3	87.9	56.3
1995–1996	91.9	76.0	94.9	76.4	85.0	59.6
1996–1997	90.4	73.3	93.4	76.7	84.5	58.0
1997–1998	88.8	69.7	92.0	76.8	82.9	56.8
1998–1999	89.0	69.5	92.3	77.6	81.3	56.3
1999–2000	86.9	65.8	91.3	77.3	80.1	56.8
2000–2001	85.3	64.5	90.1	76.4	78.9	55.8
2001–2002	80.6	59.4	–	–	–	–
2002–2003	82.7	60.3	–	–	–	–

*Birth cohorts for school year (i.e., children born October 1–September 30 of the following year).

**Figure 1:**
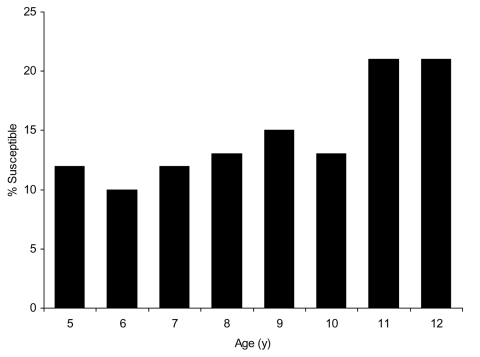
Estimates of the proportion of the population susceptible to mumps by age in 2005, applying study estimates of vaccine effectiveness to population coverage data.

Overall estimates of vaccine effectiveness were 87.8% (95% confidence interval [CI] 83.1%–91.1%) for 1 dose and 94.6% (95% CI 92.9%–95.9%) for 2 doses. There was no statistically significant variation in vaccine effectiveness for 1 or 2 doses by area of residence (p = 0.3 for 1 dose, p = 0.7 for 2 doses) or sex (p = 0.7 for 1 dose, p = 0.2 for 2 doses). Vaccine effectiveness decreased with increasing age for those who received either 1 or 2 doses (p<0.001) (Table 3). Vaccine effectiveness also varied with birth cohort (p = 0.02 for 1 dose, p = 0.003 for 2 doses). As birth cohort and age were correlated, it was not possible to separate the effects of age group and birth cohort for 1-dose effectiveness; the effect of birth cohort on 2-dose effectiveness was lost when adjusted for age. Application of our estimates of vaccine effectiveness to UK coverage data (Figure 1) predicts that >20% of children 11–12 years of age are not protected against mumps.

**Table 3:** Age-specific estimates of vaccine effectiveness for 1 and 2 doses of measles, mumps, rubella vaccine

Age group, y	Effectiveness	
	1 dose, % (95% CI*)	2 doses, % (95% CI*)
2	95.9 (81.1–99.1)	–
5–6	93.8 (84.1–97.6)	98.8 (97.0–99.5)
7–8	90.3 (81.2–95.0)	95.8 (92.9–97.5)
9–10	86.5 (75.3–92.6)	92.4 (87.7–95.3)
11–12	65.9 (30.3–83.3)	86.4 (74.1–92.9)

*CI, confidence interval; p for linear trend <0.001 for 1 and 2 doses.

### Sensitivity Analysis

Estimates of vaccine effectiveness using raw coverage data were 86.5% (95% CI 81.5%–90.3%) for 1 dose and 96.8% (95% CI 95.6%–97.7%) for 2 doses. Estimates of vaccine effectiveness increased from 87.8% to 90.8% for 1 dose and from 94.6% to 99.3% for 2 doses, for an increase in PPV of 1%–5% (Table 4).

**Table 4:** Estimates of vaccine effectiveness assuming that the proportion of the population vaccinated (PPV) was 1%, 2%, or 5% higher than reported

Coverage	Effectiveness	
	1 dose, % (95% CI*)	2 doses, % (95% CI)
Baseline	87.8 (83.1–91.1)	94.6 (93.0–95.9)
PPV + 1%	88.4 (84.0–91.6)	97.5 (96.6–98.2)
PPV + 2%	89.0 (84.8–92.1)	98.0 (97.2–98.6)
PPV + 5%	90.8 (87.3–93.4)	99.3 (99.0–99.5)

*CI, confidence interval.

**Figure 2:**
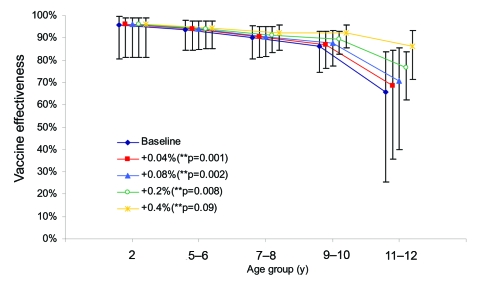
Estimates of 1-dose vaccine effectiveness for cases in 2004–05, assuming an increase in coverage of 0.04%–0.4% per year of age, which represents vaccination of approximately 1%–10% of unvaccinated persons per year of age. Values are offset on the x-axis so that 95% confidence intervals (CIs) are visible.

Age at first MMR vaccination was available for 148,525 children registered on the South Thames child health computer system. In each birth cohort, 3–45 children per year received the first MMR dose at >5 years of age, most between 5 and 6 years of age. On average, an additional 0.04% (95% CI 0.036%–0.045%) of children received MMR vaccine per year of age after their fifth birthday. In the sensitivity analysis (Figure 2), a fixed increase in coverage per year of age did not abolish the statistically significant decline in vaccine effectiveness until coverage increased by at least 0.4% per year, 10× greater than that estimated from children in the South Thames region.

## Discussion

Our estimate of 87.8% effectiveness for 1-dose mumps vaccine is lower than efficacy estimates from clinical trials (*8*,*9*) but higher than those from most published field evaluations (*10*–*14*). Lower estimates in field studies could result from problems with vaccine storage or administration, errors in case definition (i.e., clinical instead of laboratory-confirmed cases) or ascertainment, inaccurate determination of vaccination status, and bias from conducting studies during outbreaks (*10*,*28*,*29*). Because clinical trials have relatively short follow-up periods, waning immunity may also produce lower observed effectiveness in field evaluations. We observed a decline in protection with increasing age, which suggests that waning immunity may occur. After 2 doses, the magnitude of this decline is small, and effectiveness remains above 85% even 6–7 years after the second vaccination.

Research on whether protection from mumps vaccine declines with time since vaccination is contradictory. In 2 US outbreaks, children vaccinated >5 (*12*) or >3 (*19*) years before each outbreak were at higher risk for mumps. In 2 other US outbreaks, no evidence was found for increased infection rate with time since vaccination (*11*,*16*). A study from Belgium found increasing risk for disease with time since vaccination (*13*). In the presence of natural boosting, neutralizing antibodies have been demonstrated up to 12 years after vaccination (*30*). However, duration of antibody persistence in a high-coverage setting where mumps circulation has declined is not known. In Finland, a decline in mumps antibody titers was demonstrated in vaccinated children (*31*), and the proportion of children seropositive for mumps antibodies some years after MMR vaccination was lower than expected in Sweden and the United Kingdom (*32*,*33*).

Our estimates may be affected by several biases. Unvaccinated children may mix with other unvaccinated children and exposure may be more common than in vaccinated children, which would lead to an overestimation of vaccine effectiveness. Because most cases occurred in age groups not eligible for vaccination, however, it is likely that exposure to mumps was fairly uniform.

In addition, those who do not access healthcare for vaccination may be less likely to seek care for mumps disease. Consequently, these cases may be less likely to be reported, which would lead to an underestimation of vaccine effectiveness. The availability of free universal primary care, however, means that persons with mumps should not have difficulty accessing medical care in the United Kingdom. Studies during local outbreaks may also underestimate vaccine effectiveness, as a chance cluster of cases is more likely to be reported (*29*). The 2004–2005 outbreak of mumps was a national outbreak affecting all regions of the United Kingdom and is therefore unlikely to be subject to this bias (*6*).

The screening method relies on accurate estimates of population vaccine coverage (*25*). Estimates of vaccine effectiveness were similar using raw and adjusted coverage values, which suggests that our adjustments had not introduced any major bias. Sensitivity analyses that explore the effects of underestimation of vaccine coverage demonstrate that overall effectiveness could be as high as 90% for 1 dose, but this would require true coverage to be >5% higher than recorded.

If mumps cases had occurred before the period of our investigation, a false impression of waning immunity could have resulted because these cases would have affected proportionately more unvaccinated than vaccinated children (*34*). Immunity develops in children who have had mumps, so unvaccinated children would be preferentially removed from the population at risk. Therefore, with increasing age, the proportion of mumps cases diagnosed in vaccinated children would increase. From 1990 through 2003, however, UK mumps incidence was extremely low, so past infection is unlikely to be responsible for the observed decline in vaccine effectiveness with increasing age.

The possible effect of migration to and from the United Kingdom on these estimates is difficult to assess. Of immigrants to the United Kingdom, ≈80% arrive from commonwealth countries or the European Union each year, and ≈40,000 are children <15 years of age (*35*). Some of these countries do not use routine mumps vaccine; therefore, our estimates of vaccine coverage in older children may be too high. This would lead to an apparent increase in effectiveness with age, rather than the effect observed.

The use of vaccine coverage at the fifth birthday assumes that no further mumps vaccines are given after this age. If coverage gradually increased after this age, effectiveness would be underestimated for older age groups. Children 5–12 years of age, however, have generally not been targeted by MMR catch-up campaigns, and the target payment incentive in primary care does not apply after 6 years of age (*36*). From the South Thames data, most MMR given after the fifth birthday is given to children 5–6 years of age, and the numbers vaccinated are too low to produce the observed decline in effectiveness. We therefore conclude that an increase in coverage after 5 years of age is unlikely to fully explain the decline.

No evidence has shown that vaccine quality and handling changed over time. All cohorts >6 years of age were only eligible for MMR-II (Sanofi Pasteur MSD, Berkshire, UK) for the first dose, so a change in product cannot be responsible for the decline in first-dose effectiveness after this age. Priorix (GlaxoSmithKline, Uxbridge, UK) became available in 1998, but as population coverage by vaccine manufacturer is not available, we are unable to show any differences between vaccines.

According to this study, <80% of children 11–12 years of age are protected against mumps, less than the suggested threshold of 90%–92% to interrupt transmission (*37*). The true proportion of those with immunity may now be higher than this because many unvaccinated persons will have acquired natural infection and vaccinated persons may have been boosted by exposure during the outbreak.

Our estimates of vaccine effectiveness suggest that the mumps component of MMR provides excellent protection through routine administration in the United Kingdom. The observation of waning immunity is, however, a cause for concern because the proportion of susceptible adolescents may increase, which could lead to future outbreaks in this age group. Because most recent cases have occurred in persons too old to have been vaccinated, the contribution of waning immunity to the current UK outbreak is small (*6*). To minimize the risk of future outbreaks, MMR vaccine coverage with both doses must be improved and maintained.
